# Effect of Shear Stress during Processing on Structure, Morphology, and Properties of Isotactic Polypropylene Nucleated with Silsesquioxane-Based β-Nucleating Agent

**DOI:** 10.3390/ma16103627

**Published:** 2023-05-09

**Authors:** Mateusz Barczewski, Olga Mysiukiewicz, Michał Dutkiewicz, Mariusz Szołyga, Monika Dobrzyńska-Mizera, Adam Piasecki

**Affiliations:** 1 Faculty of Mechanical Engineering, Institute of Materials Technology, Polymer Processing Division, Poznan University of Technology, Piotrowo 3, 61-138 Poznan, Poland; 2 Poznan Science and Technology Park, Adam Mickiewicz University Foundation, Rubiez 46, 61-612 Poznan, Poland; 3 Faculty of Materials Engineering and Technical Physics, Poznan University of Technology, al. Jana Pawla II 24, 61-138 Poznan, Poland

**Keywords:** polypropylene, silsesquioxane, nucleation, β-phase, POSS, crystallization, processing

## Abstract

The study aimed to determine the influence of shear stress during real-life industrial processes such as compression molding and injection molding to different cavities on the crystallization of the isotactic polypropylene nucleated with a novel silsesquioxane-based β-nucleating agent. Octakis(N^2^,N^6^-dicyclohexyl-4-(3-(dimethylsiloxy)propyl)naphthalene-2,6-dicarboxamido)octasilsesquioxane (SF-B01) is a highly effective nucleating agent (NA) based on the hybrid organic-inorganic silsesquioxane cage. The samples containing various amounts of the silsesquioxane-based and commercial iPP β-nucleants (0.01–0.5 wt%) were prepared by compression molding and injection molding, including forming in the cavities with different thicknesses. The study of the thermal properties, morphology, and mechanical properties of iPP samples allows for obtaining comprehensive information about the efficiency of silsesquioxane-based NA in shearing conditions during the forming. As a reference sample, iPP nucleated by commercial β-NA (namely N^2^,N^6^-dicyclohexylnaphthalene-2,6-dicarboxamide, NU-100) was used. The static tensile test assessed the mechanical properties of pure and nucleated iPP samples formed in different shearing conditions. Variations of the β-nucleation efficiency of the silsesquioxane-based and commercial nucleating agents caused by shear forces accompanying the crystallization process during forming were evaluated by differential scanning calorimetry (DSC) and wide-angle X-ray scattering (WAXS). The investigations of changes in the mechanism of interactions between silsesquioxane and commercial nucleating agents were supplemented by rheological analysis of crystallization. It was found that despite the differences in the chemical structure and solubility of the two nucleating agents, they influence the formation of the hexagonal iPP phase in a similar way, taking into consideration the shearing and cooling conditions.

## 1. Introduction

Despite the past 70 years since polypropylene (PP) was first introduced, an interest in this material is still progressing due to its high ability to be recycled, mechanical properties allowing for a wide array of industrial applications, and easy processing [1,2]. This polymer is the second most processed thermoplastic after various types of polyethylene (low-density polyethylene LDPE, linear low-density polyethylene LLDPE, and high-density polyethylene HDPE) with a consumption in 2022 of 8.75 Mt, 16.6% of the total global consumption [3,4]. Considering the growing market share of recycled materials and insufficient market participation of biodegradable polymers, a quick replacement of this versatile polymer is not expected [3,5]. Moreover, new coupled recycling methods increase the efficiency of PP recovery which further boosts assumptions of the circular economy [6].

Polypropylene is polymorphic, hence it may crystallize in four crystallographic systems: monoclinic (α), hexagonal (β), triclinic (γ), and smectic, with the most often being an isotactic variety (iPP) [4,7,8]. In the case of products shaped via conventional molding technologies, such as extrusion, injection, or compression molding, the most frequently obtained is the α-phase—the most thermodynamically stable iPP phase. The enhanced elasticity modulus and tensile strength result from the high crystallization degree of the system. Moreover, when using selected varieties of nucleants classified as clarifiers, mainly sorbitol derivatives, it is possible to obtain increased transparency of the molded parts [9,10,11]. β-phase is thermodynamically metastable and characterized by a lower rate of primary nucleation than the monoclinic phase, which makes it more difficult to achieve in industrial processing conditions [12]. β-crystalline-dominated polypropylene presents different mechanical and processing behavior, i.e., it is characterized by improved elongation at break, impact strength, and high durability [7,13,14,15]. At the same time, it shows negative features such as lower resistance to UV radiation [16]. As classified and arranged by Papageorgiou et al. [4], the formation of the β-form of polypropylene can be obtained via a shear-induced crystallization process, direct crystallization in a temperature gradient field, quenching polypropylene from melt to a specific temperature range, an introduction of vibrations during crystallization, and addition of β-nucleating agents [4,17,18,19,20,21]. In the case of industrial practice, obtaining in a controlled way, increased content of the hexagonal phase can be achieved only by introducing β-nucleants and a certain cooling temperature profile. Since the introduction of linear trans quinacridone with γ-modification in 1967 [22], numerous β-nucleating agents have been identified and commercialized. They can be divided into categories according to their chemical composition, such as inorganic metal salts and oxides [23], amides [24], or rare earth compounds [25]. However, even the impact of effective β-nucleant may be limited when inappropriate process conditions such as intensive cooling or shear forces would be applied, as they can limit or prevent the epitaxial growth of the polypropylene hexagonal phase on the NA structures [26]. Therefore, only a part of the latest papers in this field focuses on finding novel nucleating agents [24]—understanding the relationship between the structure of the β-nucleating agent, crystallization conditions, and the resulting morphology seems to be an even more crucial goal [22,24,25].

The influence of various types of polyhedral oligomeric silsesquioxane addition on the crystallization and morphology of thermoplastic polymers, including polypropylene, was widely investigated [27,28,29,30,31,32,33,34,35]. The most commonly introduced octa-substituted silsesquioxanes functionalized with alkyl groups with various lengths, such as isobutyl, methyl, hexadecyl, or octadecyl caused only a slight change in the crystallization temperature of isotactic polypropylene (about 1–3 °C) with a negligible effect on the crystallinity level [29,30,36]. Worth mentioning is that none of the papers report the ability of alkyl-substituted silsesquioxanes to promote iPP crystallization in the hexagonal phase. As proved in the literature [37,38], the main reason for the limited application of silsesquioxanes as self-standing high-efficiency nucleating agent for polypropylene with dominated epitaxial crystallization mechanism was differences in polarity and crystallographic mismatch between silsesquioxane and iPP. At the same time, the published papers demonstrated a possibility of simultaneous use of melt-blended silsesquioxane with N,N′-dicyclohexyl-2,6-naphthalene dicarboxamide (TMB-5) without a significant loss of β-phase promotion ability. Following this line of research, several works proved that the simultaneous introduction of nucleants promoting crystallization in the forms α and β with the participation of silsesquioxane led to the formation of a reactive or physically-mixed nucleating system. As a result, it is possible to increase its effectiveness [39,40,41] or control the structure to obtain specific functional or processing characteristics of iPP [42,43,44].

The studies carried out so far have shown that the complex silsesquioxane structure may use different mechanisms of influence on the polypropylene crystallization process, depending on the conditions in which the polymer is solidified (isothermal/non-isothermal, quiescent/shear) [27,28,45]. Fu and co-workers [28] studied the crystallization process of polypropylene modified with octamethyl-silsesquioxane under shearing conditions. The research showed different mechanisms of silsesquioxane impact on iPP in steady and shearing conditions. In the case of quiescent conditions, well-dispersed silsesquioxane domains decreased crystal growth ability and limited the mobility of iPP chains, while residual silsesquioxane crystals became effective nucleating agents for iPP. For crystallization under step shear flow conditions, silsesquioxane molecules acted as physical crosslinkers limiting local relaxation times of iPP macromolecular chains and improved molecular orientation. This led to larger nuclei density resulting in increasing crystallization rate of silsesquioxane-modified iPP series.

In our previous work, we demonstrated the synthetic route and the possibility of producing the first coupled octa-substituted silsesquioxane with iPP β-nucleating ability [46]. The results showed that the silsesquioxane presence did not cause any deterioration of the nucleant efficiency, wherein the silicon-oxygen cage itself did not cause the α-nucleation, as described in the literature. However, this previous work did not consider the complex influence of crystallization conditions, which can significantly alter the growth of the hexagonal phase of polypropylene. Therefore, this study was designed to investigate the diverse and often opposite effects of shear flow, the presence of polypropylene β-phase crystallization-promoting nucleants [42], and the complex behavior caused by silsesquioxanes during polymer crystallization in shearing conditions [28]. This work presents the physical modification of polypropylene with N^2^,N^6^-dicyclohexylnaphthalene-2.6-dicarboxamide, and silsesquioxane-based NAs. The samples were shaped in the process of minimal shear impact (compression molding) and the injection molding process using molding cavities of various thicknesses, which allowed to induce of different shearing conditions during the non-isothermal flow of the material through the mold while cooling the material in order to evaluate the potential of β-nucleation in the real-life industrial conditions. To describe the silsesquioxane core impact on the supermolecular structure of β-nucleated isotactic polypropylene, the analysis of iPP structure obtained in various conditions was compared and described.

## 2. Experimental

### 2.1. Materials and Sample Preparation

#### 2.1.1. Materials and Methods

Isotactic polypropylene (iPP) with the trade name Moplen HP500N from Basell Orlen Polyolefins (Płock, Poland), with a mass flow rate (MFR) of 12 g/10 min (230 °C; 2.16 kg), was used as a polymeric matrix. The material is defined as a low modification level grade, with a molecular weight of 354.567 g/mol, and narrow molecular weight distribution, which allows for reducing the risk of limitations in the formation of hexagonal phase due to high molecular weight as it was described by Chvátalová et al. [47].

**Figure 1:**
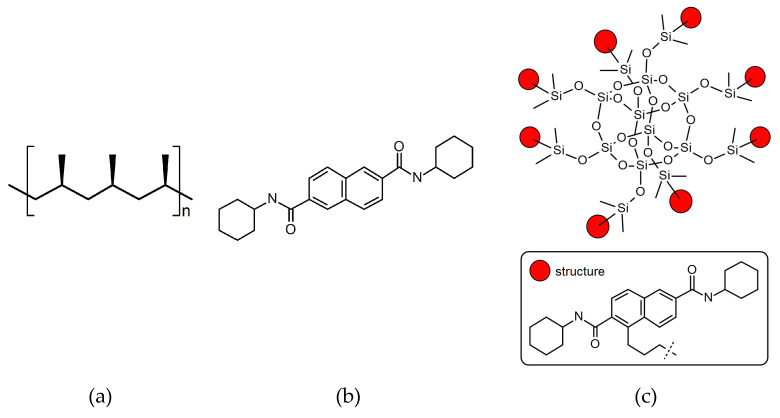
The chemical formulas of (**a**) isotactic polypropylene; (**b**) N^2^,N^6^-dicyclohexylnaphthalene-2,6-dicarboxamide; (**c**) Octakis(N^2^,N^6^-dicyclohexyl-4-(3-(dimethylsiloxy)propyl)naphthalene-2,6-dicarboxamido)octasilsesquioxane.

Two different nucleating agents were used for the modification of iPP. The first was commercial iPP β-phase nucleant, namely N^2^, N^6^-dicyclohexylnaphthalene-2.6-dicarboxamide, abbreviated NJSTAR NU-100, delivered by New Japan Chemical Co. (Osaka, Japan). The second one was silsesquioxane functionalized with NA (SF-B01), produced by the Department of Organometallic Chemistry UAM (Poznań, Poland), which synthesis and characterization were described in our previous paper [46]. As can be seen in Figure 1, SF-B01 consists of the silicon-oxygen cage and the N^2^,N^6^-dicyclohexylnaphthalene-2.6-dicarboxamide substituents, so it allows us to determine how the chemical structure would influence the β-nucleation ability of this additive. The thermal stability of all materials was determined using thermogravimetry, in the temperature range of 30–900 °C, with a heating rate of 10 °C/min in nitrogen, which confirmed the usability of these materials at temperatures usually applied during iPP processing. The chemical formulas of the nucleating agents used in our study are presented in Figure 1. The degradation temperatures of both modifiers exceed the temperature processing conditions used in these studies [48,49]. Preliminary research also showed also an insignificant effect of the use of both NAs in the applied concentrations on changes in the thermal stability of the modified polymer.

#### 2.1.2. Sample Preparation

**Figure 2:**
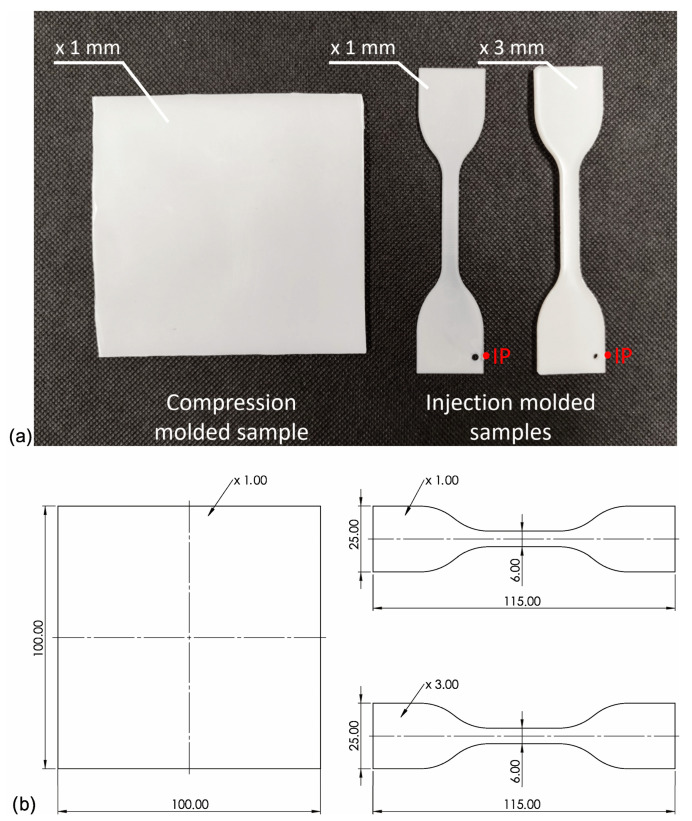
(**a**) Photography of the samples used in the experiment formed by compression and injection molding, IP—injection molding gate; (**b**) Dimensions of the compression molded and injection molded samples.

Before mixing in a molten state, iPP pellets were milled into powder in a Tria high-speed grinder. Then iPP and NAs powders were premixed using a high-speed rotary knife grinder Retsch GM200 (*t* = 3 min. *n* = 3000 rpm) with different amounts of nucleating agents (0; 0.01; 0.05; 0.1; 0.25; 0.5 wt%). Next, all blends were mixed in a molten state using a Zamak16/40 EHD twin screw extruder operated at 190 °C and 100 rpm and pelletized after cooling in a water bath. To differentiate the shearing conditions during the processing of the samples, the specimens were prepared using three methods: compression molding (which can be attributed to very low shearing) and injection molding to 3 or 1 mm molds (moderate and high shearing, respectively). The injection molded specimens were prepared with an Engel ES 80/20 HLS injection molding machine (Schwertberg, Austria). The injection molding process was realized with the following parameters: mold temperature *T*_mold_ = 50 °C/min, injection speed *V* = 100 mm/s, forming pressure *P*_f_ = 5.5 MPa, and cooling time *t* = 30 s. Compression molding was carried out using a Fontjine LabManual 300 hydraulic press, using a steel frame with a forming cavity of 100 × 100 × 1 mm between two PTFE sheets. The high-pressure compression molding process was performed at the temperature of 190 °C and a forming time of 5 min, and then, after removal from the working space of the press, it was freely cooled under a load of 10 MPa. The physical appearance of the samples has been presented in Figure 2. The melt temperature has a distinct effect on the iPP supermolecular structure of nucleated with β-nucleant. Therefore, the relatively low temperature of the melt was used for both processing technologies (190 °C) to limit the effects of α-phase formation [47]. The entire injection-molded system, together with a sprue, was presented in the work [50].

### 2.2. Methods

To evaluate the impact of both NAs on the polypropylene crystallization, the samples were investigated using differential scanning calorimetry (DSC). Samples of 5 ± 0.2 mg, placed in aluminum crucibles with pierced lids, were subjected to heating in the range of 20–230 °C (10 °C/min), then held at the final temperature for 5 min and cooled back to 20 °C (10 °C/min). The analysis was performed using an F1 204 Phoenix apparatus operating in a nitrogen atmosphere. The heating was performed twice. The first scan was used to analyze the influence of the processing parameters (shear stress) on the crystalline structure of polypropylene, and the second one—was to evaluate the structure formed in quiescent conditions. The β-phase content (*Φ)* was determined following Equation (1):

(1) $$\Phi =\frac{X_{\beta }}{X_{c}}\cdot 100\%=\frac{X_{\beta }}{X_{\beta }+X_{\alpha }}\cdot 100\%$$

where: *X_β_*—the β phase crystallinity, *X_α_*—the α phase crystallinity, *X_c_*—total sample crystallinity.

The crystallinity of the *i* phase *X_i_* was calculated in the following way (2):

(2) $$X_{i}=\frac{∆H_{i}}{∆H_{i,100\%}}\cdot 100\%$$

where Δ*H_i_*—melting enthalpy of the *i* phase, Δ*H_i,_*_100%_—melting enthalpy of 100% crystalline *i*, Δ*H_i_*_,100%_ = 178 J/g for *i* = α and Δ*H_i_*_,100%_ = 170 J/g for *i* = β [4].

**Figure 3:**
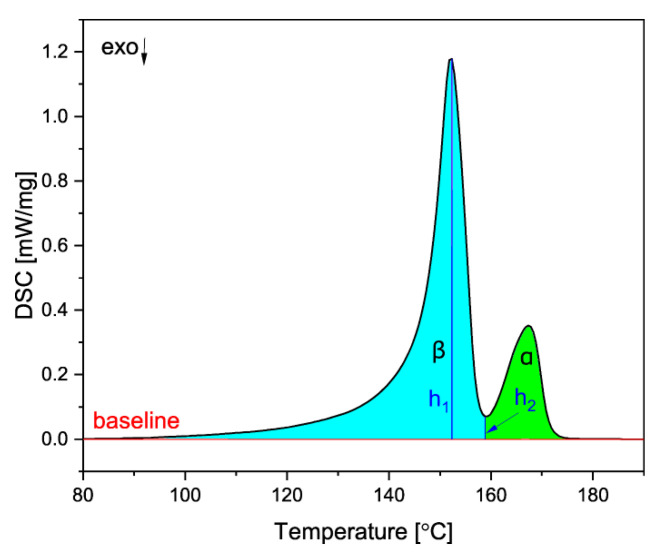
The method used to obtain the melting enthalpies of both crystalline phases.

Because the melting of the α and β crystals overlap, a special procedure proposed by Li and Cheung was used to obtain the values of the heat of fusion [51]. To calculate the melting enthalpies of the two phases, a baseline was constructed on the DSC curve, and the melting peak was divided into two areas: α(ΔH_α_*) and β(ΔH_β_*). Then, the values of Δ*H_β_* and Δ*H_α_* were obtained according to the Equations (3)–(5):

(3) $$∆H_{\beta }=A\cdot ∆H_{\beta }^{*}$$

(4) $$A={\left(1-\frac{h_{2}}{h_{1}}\right)}^{0.6}$$

(5) $$∆H_{\alpha }=∆H_{c}-∆H_{\beta }$$

where Δ*H_c_* is the combined melting enthalpy of the two phases, obtained from the area under the DSC curve of the two peaks, *h*_1_ is the β melting peak height and *h*_2_ is the height from the baseline to the minimum between the α and β peaks, as shown in Figure 3.

Wide-angle X-ray diffraction (WAXS) analysis was undertaken using a Bruker D2 PHASER apparatus (Billerica, USA) with XFlash^®^. A monochromatic X-ray radiation with a wavelength of *λ* = 1.5406 Å (Cu_Kα_) was used. A reflected X-ray peak intensity analysis at a defined 2*θ* angle was used for identification purposes. For injection molded samples, measurements were made for the gate side of the specimen, perpendicular to the flow direction of the polymer in the mold. For the description of the iPP monoclinic phase, the following crystallographic planes were analyzed: (110), (040), (130), (111), and (130), reflected by 14.1°, 16.9°, 18.8°, 21.2°, and 22° diffraction angles. For the iPP hexagonal phase, (300) and (301) crystallographic planes measured at diffraction angles of 16.2° and 21.2° were analyzed. To calculate a β-phase content in a crystalline iPP matrix, the Turner-Jones Equation (6) was applied [50]:

(6) $$k=\frac{I_{\beta (300)}}{I_{\alpha 1(110)}+I_{\alpha 2(040)}+I_{\alpha 3(130)}+I_{\beta (300)}}\cdot 100\%$$

where: *k* value is a calculated percentage hexagonal phase content in crystalline iPP, *I_β(300)_* is a diffraction peak intensity determined for (300) crystallographic plane, while *I_α_*_1(110)_, *I_α_*_2(040)_, *I_α_*_3(300)_ are the diffraction peaks intensities measured at (110), (040) and (130) planes, accordingly. The evaluations proceeded according to the two-phase concept [23,49,50,51,52]. Moreover, in the separation of the crystalline fraction from the amorphous halo, the Gauss functions with a nonlinear regression procedure were applied [52].

The MCR 301 Anton Paar rheometer (Graz, Austria) operating in the 25 mm plate-plate configuration, using a gap of 0.5 mm, under the oscillatory shearing mode with an angular frequencies ω of 0.1, 1, and 10 rad/s and a strain of 1% was used in the rheological investigations. Polymeric samples were heated up to 200 °C and held at this temperature for 5 min to erase thermal effects. After annealing, molten samples were cooled with a cooling rate of 5 °C/min in the presence of shear in order to describe the changes between samples in crystallization behavior. Material from injection molded samples with a thickness of 1 mm was taken for testing.

The analysis of the microstructure of polymeric samples was realized using scanning electron microscopy (SEM) by means of a Tescan MIRA3 microscope (Czech Republic, Brno—Kohoutovice). The measurements were conducted with an accelerated voltage of 5 kV and magnifications of 200×, 1000×, and 10,000×. The analysis was performed with an accelerated voltage of 12 kV in backscattered electrons (BSE) and the secondary electron (SE) mode. The carbon coating (∼20 nm) was deposited on samples using the Jeol JEE 4B vacuum evaporator (Jeol, Tokio, Japan).

The mechanical performance of polymeric samples was evaluated in a static tensile test. The measurements of yield strength, elastic modulus, and elongation at break were considered. The tensile tests were performed per ISO 527 using Zwick/Roell Z020 5101 universal testing machine (Zwick Roell Group, Ulm, Germany). The measurements were conducted at room temperature. The elastic modulus evaluation was realized at a cross-head speed of 1 mm/min, and a 50 mm/min elongation speed was used for the other part of the experiment. The presented in this study values are the mean results of nine samples for each series.

## 3. Results and Discussion

**Figure 4:**
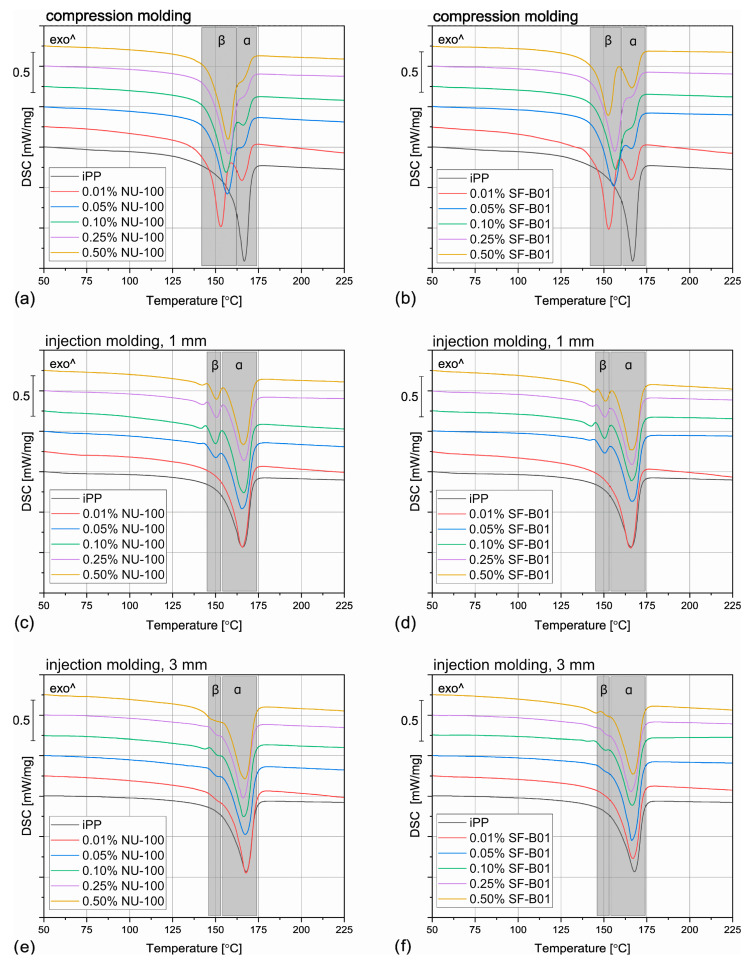
The DSC thermograms obtained during the first heating of the samples nucleated with different amounts of NU-100 and SF-B01 processed by (**a**,**b**) compression molding, (**c**,**d**) injection molding into 1 mm mold, and (**e**,**f**) injection molding into the 3 mm mold.

**Figure 5:**
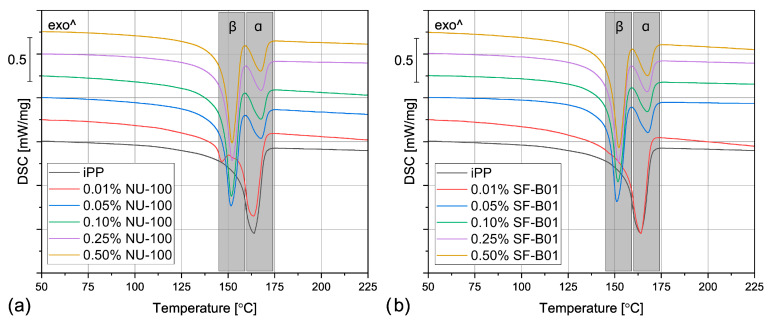
The DSC thermograms obtained during the second heating of the samples nucleated with different amounts of (**a**) NU-100 and (**b**) SF-B01, injection molded into the 1 mm mold.

**Figure 6:**
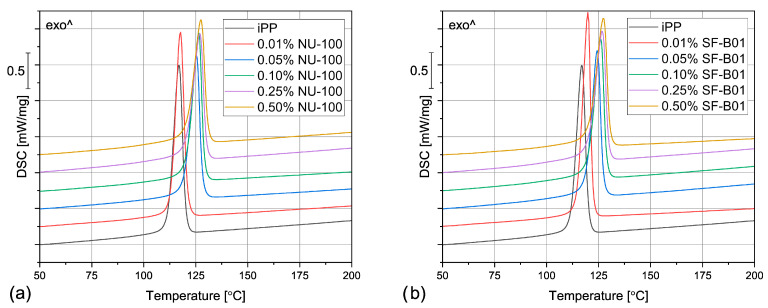
The DSC thermograms obtained during cooling of the samples nucleated with different amounts of (**a**) NU-100 and (**b**) SF-B01, injection molded into the 1 mm mold.

The DSC thermograms obtained during the first heating of the nucleated samples and iPP formed in different conditions are presented in Figure 4. The exemplary curves obtained during the second heating and cooling of the 1 mm injection molded specimens are shown in Figure 5 and Figure 6, respectively. The corresponding thermograms of the samples processed in different conditions were very similar, and they can be found in the Supplementary Materials (Figures S1 and S2). The results of the first and the second DSC scans, such as the α and β phases melting temperatures (*T_mα_*, *T_mβ_*), the crystallization temperatures *T_cr_*, the β-phase content *Φ* and the total crystallinity *X_c_* are collected in Table 1 and Table 2.

To evaluate the influence of the nucleating agents on polypropylene crystallization, the second DSC run was analyzed showing the structure crystallized in controlled, quiescent conditions without shearing. As can be seen in Table 2 and Figure 5, all the samples nucleated with NA content of 0.05 wt% and higher show two melting peaks centered around 151 °C and 167 °C, resulting from the β and α phases melting, respectively. Therefore, it was confirmed that both commercial NU-100 and novel SF-B01 were efficient β-phase nucleating agents, with increasing ability to induce the crystallization of the hexagonal phase with the content. It was also observed that the addition of 0.01 wt% of either NU-100 or SF-B01 did not result in the formation of the β phase (or, presumably, the *Φ* value is too low to be detected using the thermal analysis). When it comes to the samples containing higher concentrations of NA, regardless of the nucleant used and the processing procedure, the calculated β-phase content is in the range of 71–81%, which is in line with the results of our previous research on this subject [46]. The differences can be attributed to the changes in the NA dispersion and the inevitable simplifications during the signal processing and calculations according to Li and Cheung’s method. Interestingly, all the nucleated samples crystallized in quiescent conditions also present an elevated total crystallinity degree—about 50–55% in comparison with 40% obtained for iPP, which further confirms NAs efficiency. It can be also seen that 0.01 wt% of NU-100 or SF-B01 may not result in successful β nucleation, but it increases the total crystallinity of polypropylene.

Even though the amount of the filler above 0.01 wt% did not visibly influence the total crystallinity and the β phase content, it does play a vital role in the polypropylene crystallization, as it is indicated by the results of the DSC cooling step. In Figure 6 and Table 1, it can be observed that the unfilled polypropylene crystallizes at 117 °C. Even the relatively low NU-100 content of 0.05 wt% increases this value by 8 °C and the addition of 0.50 wt% of this NA shifts *T_cr_* even more, to 127 °C. The silsesquioxane-based nucleating agent causes a similar increase in the iPP crystallization temperature. These results indicate that the nucleated samples can form the crystalline phase at a lower undercooling. It can be therefore presumed that both additives act as crystallization centers, however, this claim should be also confirmed by full crystallization kinetics analysis in further research. Nevertheless, the observed increase in *T_cr_* is highly beneficial from the practical point of view, as it results in shorter production cycles.

When the physical modifier type, its content as well as the processing history are considered, the samples present a more complicated behavior, which can be observed in Figure 4 and Table 1. The compression molded samples show a similar thermogram as the materials crystallized in quiescent conditions, with prominent β-phase peaks. As the shear rate during this method is almost negligible, this result is understandable. However, the conditions during crystallization in highly-controlled conditions in the DSC furnace and during free cooling after compression molding are not the same, especially when it comes to the cooling rate, so differences should be expected. The most prominent difference can be observed for the samples with 0.01 wt% of the NA, which in this case reveal similar β-phase content as the remaining nucleated samples. It can be presumed that during compression molding the samples were held at an elevated temperature for a shorter time, so the crystallization of the α phase was limited. What is more, in comparison with the samples crystallized in the DSC pan, the *T_mβ_* is shifted to slightly higher temperatures around 157 °C, which indicates that more perfect or larger hexagonal phase crystals were formed during the compression molding. On the other hand, in this case, the calculated *Φ* values are lower and they also depend on the modifier content. For example, the 0.05% NU-100 sample presents a β-phase content of 64%, and the 0.25% NU-100—73%. The specimen with the highest NA content shows the *Φ* value of 72%. An even more interesting phenomenon was observed in the case of the SF-B01-modified samples, where the highest *Φ* value of 71% was calculated for the composition with 0.25 wt% of the NA, whereas the remaining specimens obtain the β-phase content of around 60%. It can be stated that either too low or too high NA content can be disadvantageous—when it does not exceed 0.10 wt% the amount of the crystallization seeds is too low to obtain higher *Φ* values and its percentage of 0.50 wt% results in agglomeration of the NA particles, which also decreases its efficiency [52].

During injection molding to the 1 mm thick mold, the material is subjected to the highest shear rate of all the examples described in this paper. Nevertheless, for iPP only the melting of α-crystals was recorded. It is known that shearing promotes β-phase crystallization in non-nucleated polypropylene [53], however, as shown by Zhang et al., the processing temperature of 190 °C (applied in the study) may limit the formation of the hexagonal phase [20]. The modified samples show three partially overlapping endothermic peaks: around 143 °C, 151 °C, and 166 °C. The first one can be identified as the so-called annealing peak, which is an effect of the enthalpy recovery of the rigid amorphous fraction (RAF) [54]. This result can be surprising, as the samples were not subjected to a separate annealing step; however, this phenomenon may be an effect of the complex cooling condition in the center part of the injection molded sample. Interestingly, this behavior was also recorded for the 0.01% NU-100 sample crystallized in quiescent conditions. It can be hypothesized that in certain cooling conditions when the hexagonal phase cannot be fully formed because of extensive shearing or insufficient cooling rate, the presence of the β nucleating agent leads to an increase in the RAF amount. The remaining peaks can be attributed to the melting of the α and β phases. In comparison with the compression molded samples, the β melting peak is much less prominent, which correlates with the obtained *Φ* values below 22%. In the case of the 0.01 wt% NU-100 and 0.01 wt% SF-B01, the hexagonal phase could not be detected. This result can be explained by an increased shearing during sample processing. Unlike in the case of the unmodified polypropylene, in the presence of a nucleating agent, the β phase formation is hindered by shearing, which inhibits the development of the isochiral-rich mesophase needed for β crystallization [55]. A clear relationship between the hexagonal phase content and the NA amount is observed. It can be observed, that the addition of 0.05 wt% of either NU-100 or SF-B01 results in noticeably lower *Φ* values, whereas the differences between the remaining samples are negligible. It may be concluded that when crystallization takes place in disadvantageous conditions, a minimum of 0.10 wt% of the nucleating agent is needed for efficient crystallization.

The samples injection molded in the 3 mm molds show similar behavior as their 1 mm thick counterparts, but in their case, the β-phase content is even lower. In the majority of the specimens, only the presence of the α crystals was confirmed and the *Φ* values obtained 0.05% NU-100, 0.10% NU-100, and 0.10% SF-B01 were below 3%, so they are negligible. These results may seem counterintuitive as in this case, the shearing was less intensive in comparison with injection molding into 1 mm mold. It can be explained when cooling conditions are taken into consideration. Even though the two types of injection molded samples were prepared with the same mold temperature, it is quite obvious that the cooling of a 3 mm sample is slower in comparison with the 1 mm one, especially in the specimen’s center. Therefore, the thicker samples stayed longer at the higher temperature, which promoted the growth of the α crystals [56]. To obtain injection molded polypropylene parts characterized by relatively thick walls and high β-phase content, a more efficient cooling method needs to be implemented.

**Figure 7:**
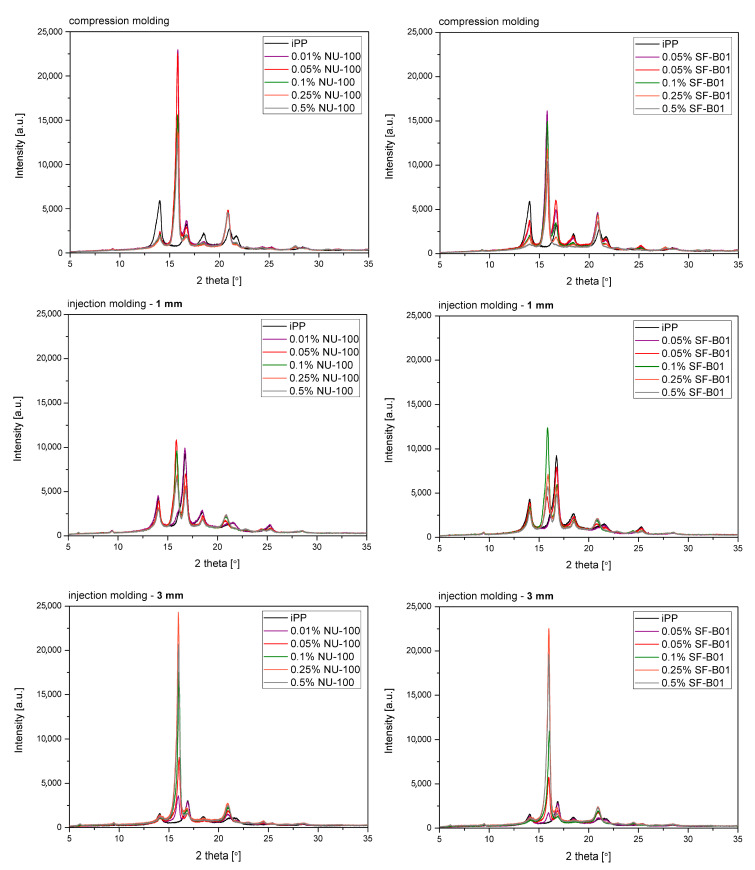
WAXD diffractograms of iPP nucleated with NU-100 and SF-B01 formed in various shearing conditions.

**Figure 8:**
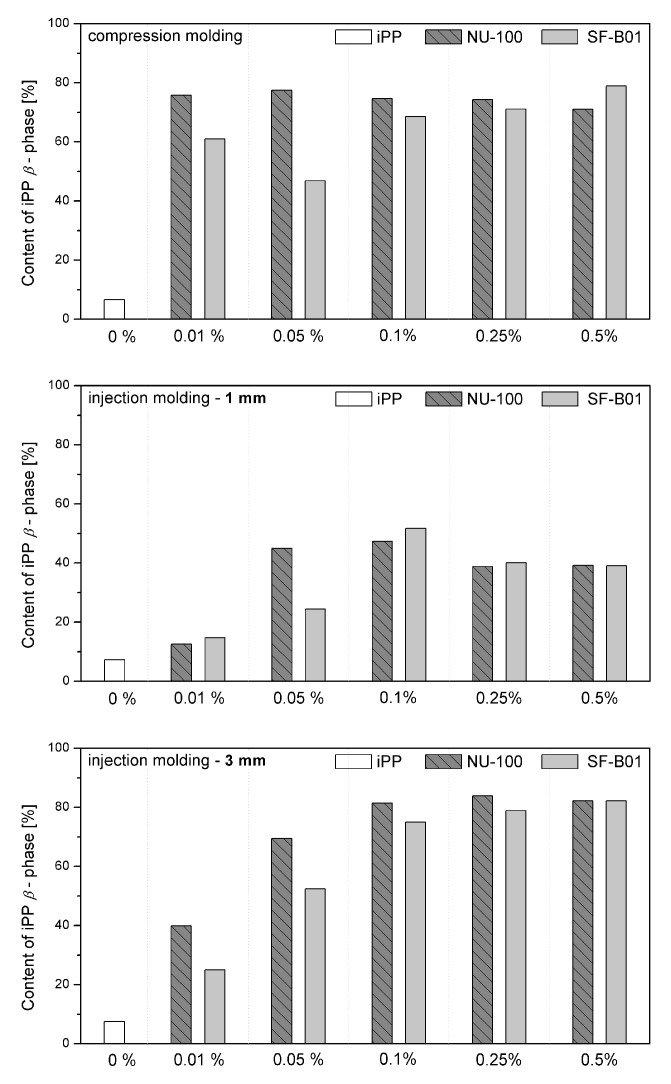
WAXD-calculated content of iPP β-phase in samples formed with various processing techniques.

Figure 7 shows the WAXD diffraction patterns of pure polypropylene and the one modified with NAs, shaped in the compression and injection molding processes. Calculated from WAXD data, based on Equation (6), β-phase contents are summarized in Figure 8. In the considered case, the process conditions slightly impacted the content of β-phase in polypropylene samples, revealing its comparable low values. The highest value (7.5%) was noted for the 3 mm injection molded sample, while the lowest (6.6%) was for compression molded iPP series. Despite the use of a relatively high mold temperature (50 °C), based on the results of 1 mm samples, it can be stated that the cooling rate had a dominant effect on the crystallization process. According to Nezbedowa et al. [57], the β-phase content obtained for products shaped in the injection process depends not only on the shear rate, related to the injection speed but also on the temperature of the mold. While in the presence of a β-nucleant, increasing the injection rate results in an increased share of the hexagonal form. On the other hand, lowering the mold temperature significantly reduces this phenomenon. In the considered case, despite the higher values of shear forces, the intensive heat exchange must have limited influence on the shear rate during the formation of the β-phase (1 mm injection molded series). When comparing a 1 mm with a 3 mm injection molded β-nucleated series, higher hexagonal phase content was noted for 3 mm samples. The obtained results are consistent with the data presented in the work of Huo et al. [58]. Reduced content of β-phase characterized polypropylene series shaped with increased shear. The introduction of intensive shear rates during crystallization accelerates the growth of iPP α-crystals. It should be underlined that the presence of shearing forces during the crystallization of polypropylene modified with a nucleant led to the promotion of hexagonal phase formation; however, its effectiveness would be lower. This is due to the limitations of the lying of the nucleating agent on the nuclei surface mechanism. Considering the effects of creating physical interactions during the shear flow between iPP and silsesquioxane, described by Fu et al. [28], under high shear conditions, the presence of SF-B01, despite the functional groups with the ability to form β-phase, could cause an increased density of nuclei resulting in crystallization of α-phase. Among the unmodified polypropylene samples, the highest content of β-phase was recorded for the series injected in a mold with a cavity thickness of 1 mm and the lowest for the compression molded sample. This effect is consistent with literature data [20,59,60,61] and proves the occurrence of the shear-induced β-crystalline phase growth effect. The lower content of the β-phase observed for the sample formed without shearing during cooling (compression molded) may result from the slow cooling of the material and the monoclinic phase’s nuclei formation above the optimal temperature range promoting the formation of the hexagonal phase. The higher cooling rate of the 3 mm specimen made it possible to achieve favorable conditions for the growth of the β-phase, with a minor negative impact of shear forces on the effectiveness of the interaction of both nucleants. For all material series, silsesquioxane-cored NA shows lower efficiency in promoting β-crystals formation than NU-100 for the series containing 0.05 wt% concentration of NA. At the same time, the crystalline phase content was significantly lower for the compression molded sample and 1 mm, which may result in the first case from poorer dispersion of the insoluble in the iPP matrix silsesquioxane derivative. The sample formed in high shearing and rapid cooling conditions by the mechanism mentioned above of blocking macromolecules through the branched structures of the silicon cage favors the effect of solidification of the polymer structures in the boundary layer without the possibility of obtaining an oriented structure. In the case of NAs concentrations of 0.25 and 5 wt%, determined based on WAXD, the content of β-phase is comparable, regardless of the method of formation, which proves that the nucleation ability of the additive does not increase with its content in a linear way, as it was also shown in the DSC analysis, where the highest β-content values were obtained in the case of the samples containing 0.25 wt% of either SF-B01 or NU-100. It is, therefore, possible to obtain not only reactive nucleating systems as was previously mentioned in the literature but also coupled systems based on silsesquioxanes conjugated with β-nucleants, which despite the nature of silsesquioxane will fully transmit the impact of the applied functional group, regardless of the conditions during forming and solidification.

**Figure 9:**
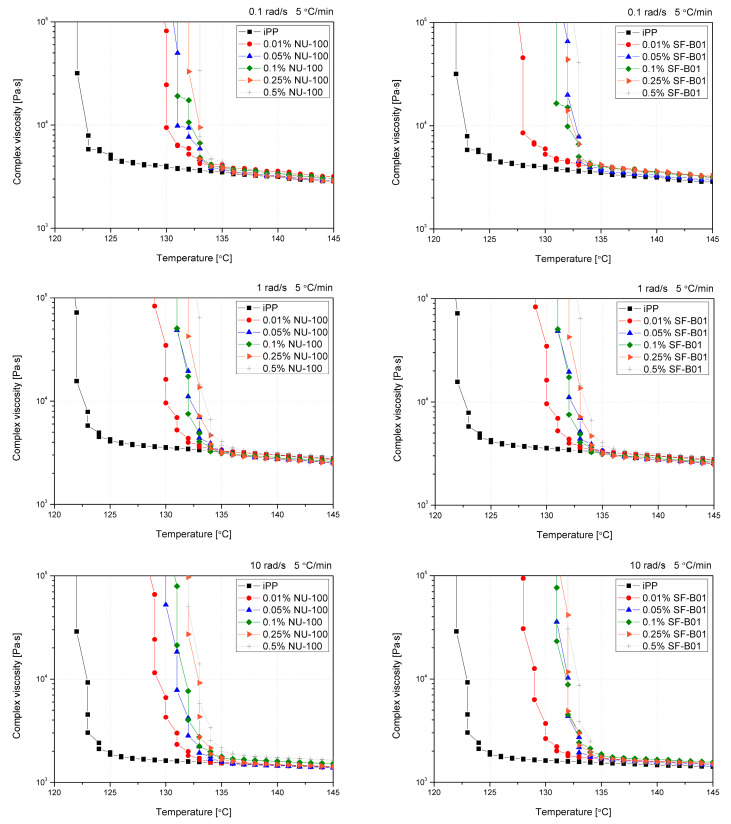
Viscosity vs. temperature curves obtained for pure and nucleated iPP cooled with 5 °C and 0.1, 1, and 10 rad/s shearing.

The oscillatory rheological measurements allow the determination of the polymer crystallization temperature with simultaneously applying the shearing conditions of various intensities. In previous studies, this method was mainly used to evaluate changes in the course of crystallization of polypropylene modified with sorbitol derivatives [40,41,44], which was associated with a formation of physical nucleant network during cooling, on which α crystallites were built up. However, Luo et al. [62] used this method to indirectly assess changes in the course of crystallization of β-nucleated iPP by WGB nucleant. Figure 9 shows the curves of complex viscosity (|η*|) changes as a function of temperature for iPP with various contents of NAs cooled at different shearing conditions with a constant cooling rate (5 °C/min). Assuming comparable cooling conditions with those used during DSC measurements, obtaining a rich β-phase structure is possible. As described by Varga and Menyhard [63], the mechanism of crystallization of iPP modified with NU-100 is related to the recrystallization of needle-like crystalline domains on which β- or α-phase of polypropylene will grow, depending on the cooling conditions connected with a critical temperature of the β-iPP formation. The tests were aimed at verifying the possibility and susceptibility of iPP modified with both nucleants to form β-phase under shearing conditions. Non-nucleated iPP reveals simple thermo-rheological (Arrhenius) behavior [62], which caused a simple one-step complex viscosity increase after starting the crystallization process. The introduction of NAs caused significant changes at the beginning of the viscosity increase process, resulting from the forming crystallization sites associated with active nucleation centers consisting of recrystallized NU-100 crystals or dispersed SF-B01 domains. In the case of the lowest concentration of NAs (0.01 wt%), a two-stage increase in viscosity can be observed for all the angular frequencies used. Considering the small share of NAs, the first increase in viscosity observed was related to the β-phase formation. In the case of increasing angular frequency (ω), this effect is gradually weakened, which can be connected with the limiting impact of shear forces on the effectiveness of β-nucleants efficiency [58]. For the lowest values of ω (0.1 rad/s), the phenomenon of complex viscosity increase associated with the formation of a complex crystalline system was noted for series containing up to 0.1 wt% NU-100 and 0.25 wt% SF-B01. This phenomenon is completely limited in the case of NU-100 for ω of 10 rad, while in SF-B01, it can still be observed for the selected series. This may indicate a lower susceptibility of the complex nucleating system based on an silsesquioxane core to the influence of shearing conditions during non-isothermal crystallization.

**Figure 10:**
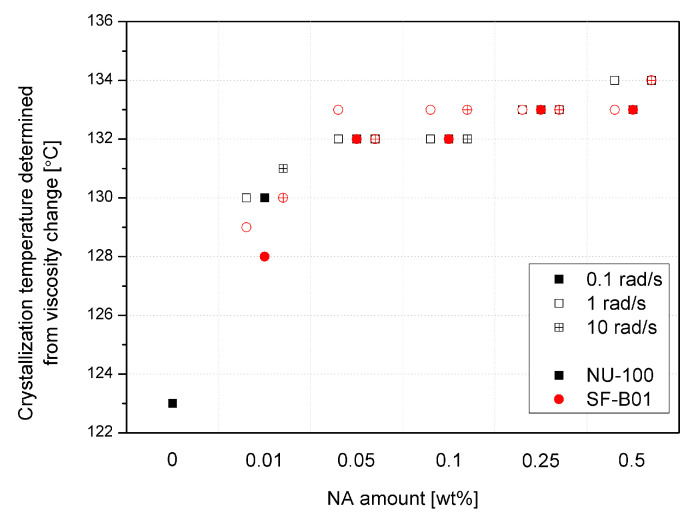
Crystallization temperature determined from rheological data.

Crystallization temperatures, determined by the rheological method based on a 20% increase in viscosity from the point of inflection of the first derivative of the complex viscosity signal, are shown in Figure 10. Even though the reported values are higher than in the case of the DSC analysis (which results from different physical phenomena taken into consideration, which are exothermic reactions in the case of DSC and viscosity increase in the case of the rheological measurements), both methods show the increase in the crystallization temperatures in the function of the nucleating agents’ content. The viscosity increase temperature of the composition caused by the separation of the crystalline phase in the additive range of 0.05 to 0.5 wt% does not differ significantly between the additives and NAs content used, as it was also reported in the case of the crystallization temperature determined by thermal analysis. Only in the case of the lowest content (0.01 wt%) the lower effectiveness of SF-B01 was noted. It is related to the more prominent two-stage viscosity increase effect observed in the |η*|(*T*) curves, probably associated with the crystalline build-up phase on functionalized silsesquioxane domains not dissolved in the iPP matrix.

**Figure 11:**
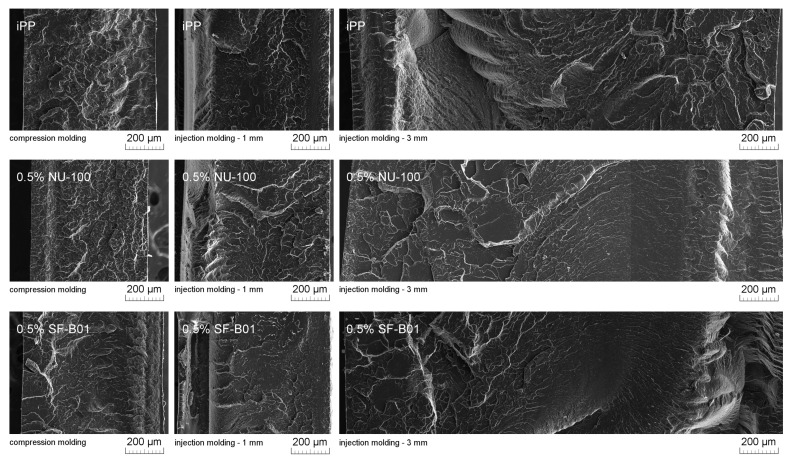
SEM images of the brittle-fractured cross-section of iPP, 0.5% NU-100, 0.5% SF-B01.

**Figure 12:**
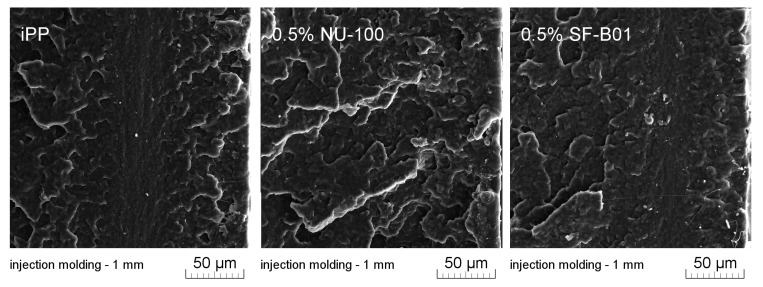
SEM images of 1 mm thick injection molded samples of brittle fractures, the external layer at the right side.

**Figure 13:**
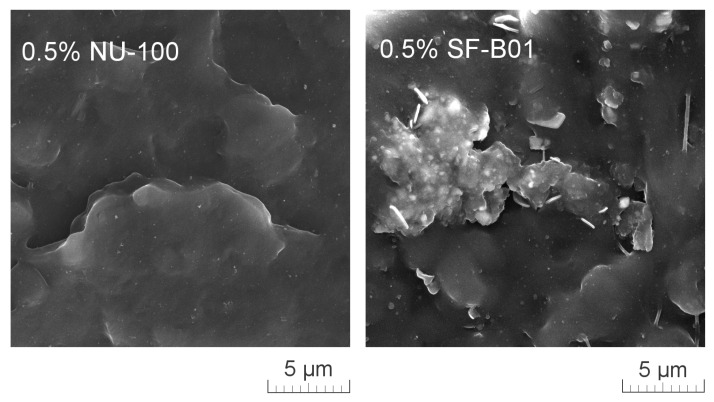
High magnification SEM image of 0.5% NU-100 and 0.5% SF-B01 injection molded 1 mm brittle fracture.

Figure 11 presents SEM images of brittle fractures of polymer samples (iPP, 0.5% NU-100, and 0.5% SF-B01) showing their entire cross-section. The most significant differences in the fracture characteristics can be noted for samples formed at high shear rate values (injection molding—1 mm), whose approximate near-edge layer was shown in SEM images made with higher magnification (Figure 12). In the case of these samples, the structural gradient in the boundary layer, described in the literature [64,65], is observable for the non-nucleated iPP and the sample containing silsesquioxane-derived nucleant. Taking into consideration the nature of the fracture of injection molded samples, an analogy can be found to the one described by Karger-Kocsis and Friedrich [64] skin-core morphology of polypropylene samples. However, the 0.5% NU-100 sample is characterized by the lack of a distinct range of the boundary layer, as is the case for the other series analyzed in Figure 11 and Figure 12. The nature of the fracture of slowly cooled compression molded samples can be related to the results of DSC measurements and the observations of Kersh et al. [65] concerning the fracture structure of polypropylene with a dominant share of α- and β-phases. In the case of samples containing 0.5 wt% of both nucleants, a much more complex fracture structure was observed compared to non-nucleated iPP. Figure 13 shows the breakthrough of samples containing the highest concentration of both nucleants used, which allows us to understand the different interaction mechanisms of both compounds. In the case of the modified NU-100 system, nucleant particles are invisible. Conversely, silsesquioxane tends to crystallize in the form of lamellar precipitates that can form microscopically observable structures in the polymer matrix.

**Figure 14:**
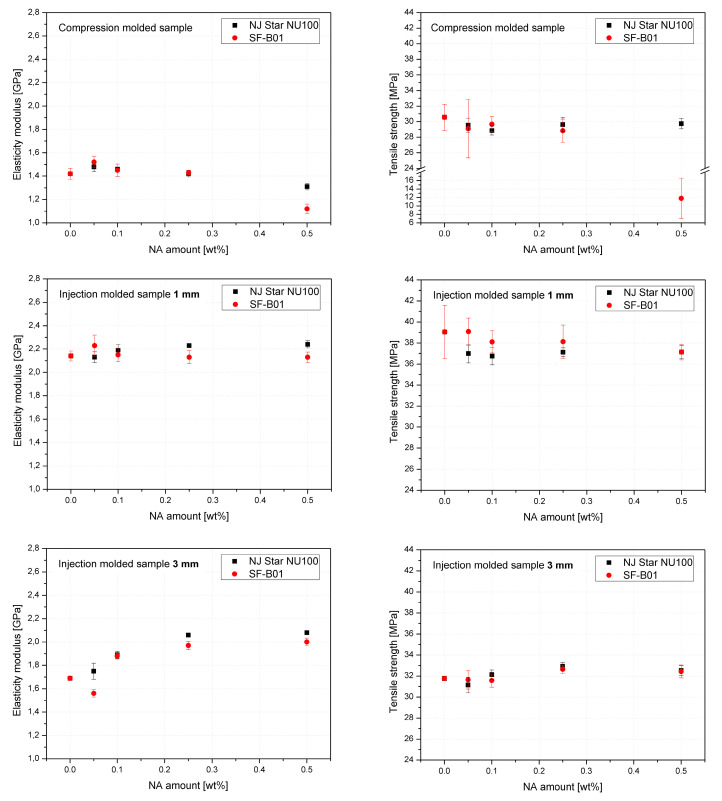
Mechanical properties of iPP modified by NU-100 and SF-B01 formed in various shearing conditions.

Figure 14 shows the mechanical properties results. The values of elasticity modulus and tensile strength as a function of NAs concentration were presented on separate charts for 3 series formed in different conditions. The highest and most beneficial effect of the addition of nucleants on the improvement of stiffness was noted in the case of 3 mm injection molded samples, which can be attributed to the increased total crystallinity and the lack of the less stiff β phase, as determined by DSC. In the case of samples formed with the minimum shear forces (compression molding) and the largest (1 mm), introducing both types of nucleants resulted in a deterioration of tensile strength. Similar results were obtained by Luo et al. [62] for iPP modified with β-nucleant trade name WBG-II. However, it should be noted that a significant deterioration in the strength of the compression molded 0.5% SF-B01 sample concerning the others may result from the creation of dispersed in polymeric matrix crystalline microscopic domains of silsesquioxane structure as presented in Figure 13, and a change in the interaction mechanism, which in the case of NU-100 is related to the partial solubility of the nucleant in the polypropylene matrix [63].

## 4. Conclusions

This research studied the influence of polypropylene shaping conditions on the crystallization behavior of β-nucleated polypropylene. As part of the considerations, the effectiveness of the commercial nucleant N^2^,N^6^-dicyclohexylnaphthalene-2,6-dicarboxamide (NU-100) was compared with its silsesquioxane analogue (SF-B01).

Functionalization with β-nucleant silsesquioxane core made it possible to limit the characteristic effects of the silsesquioxanes’ impact on the iPP structure described in the literature. It was found that the addition of the novel SF-B01 resulted in the formation of up to 80 % β-phase content in quiescent conditions, which was comparable with the results obtained for the commercial NU-100. Moreover, the crystallization of the iPP-SF-B01 system under shearing conditions does not cause the orientation changes described in the literature concerning silsesquioxane with alkyl substituents due to the physical impact of silsesquioxane on macromolecules, promoting α crystallization to a greater extent. Both types of samples subjected to the most intensive shearing during injection molding to 1 mm thick cavities were characterized with up to 20 % hexagonal phase content. It was also found that the cooling rate during crystallization also had a noticeable impact on the β-nucleating ability, which resulted in limited content of the hexagonal phase formed in the core of the 3 mm thick samples, despite the favorable low shearing. This was also the reason behind the apparent discrepancies between the results of the crystallinity analysis performed by DSC and WAXD, which in fact, showed the differences in the β-content in the core of the samples and at the surface. The rheological analysis of the crystallization process under shearing conditions allowed us to conclude that the substitution of the nucleant with silsesquioxane functional group limits the shear forces impact at the beginning of nucleation of the hexagonal phase of PP. Based on the comprehensive analysis, the differences in the behavior of both nucleants result from the change in starting point of the nucleation mechanism as silsesquioxane-based nucleant is insoluble in a polymer matrix, contrary to NU-100, which was also confirmed by the SEM observations.

The presented results showed that despite the differences in the chemical structure and solubility, both nucleating agents have a similar influence on the crystallization of iPP and also on their mechanical properties—the addition of 0.5 wt% of NU-100 or SF-B01 caused a 23 % increase in the elasticity modulus of the 3 mm samples. Both types of nucleated specimens also crystallized at about 10 °C higher temperatures than the unmodified iPP, which can significantly shorten the industrial production processes. Therefore, it can be concluded that oligomeric silsesquioxanes functionalized with the proper substituents can serve as β-nucleating agents comparable with the commercial ones.

## Figures and Tables

**Table 1:** The thermal properties of iPP and the nucleated samples obtained during the first heating and cooling.

	Sample	*T_mβ_* (°C)	*T_mα_* (°C)	*T_cr_* (°C)	*Φ* (%)	*X_c_* (%)
Compression molded	iPP	-	166.7	117.6	0	39.2
	0.01% NU-100	153.2	165.4	117.6	64.3	54.3
	0.05% NU-100	156.9	166.8	125.0	64.2	52.8
	0.10% NU-100	156.2	166.0	125.9	66.4	52.3
	0.25% NU-100	157.6	167.6	126.8	72.9	52.7
	0.50% NU-100	157.3	167.2	127.0	71.8	53.7
	0.01% SF-B01	152.8	165.9	118.7	69.5	55.7
	0.05% SF-B01	155.7	166.0	122.3	57.5	54.1
	0.10% SF-B01	156.9	167.1	125.6	59.5	53.8
	0.25% SF-B01	156.4	167.2	126.7	70.9	53.0
	0.50% SF-B01	152.5	166.2	127.2	60.3	48.8
Injection molded, 1 mm	iPP	-	165.9	116.9	0	36.6
	0.01% NU-100	-	165.8	117.7	0	47.4
	0.05% NU-100	150.1	165.2	125.3	7.9	47.6
	0.10% NU-100	150.0	166.5	126.4	17.9	47.8
	0.25% NU-100	150.5	166.4	127.0	18.1	47.8
	0.50% NU-100	150.6	166.2	127.5	19.5	41.1
	0.01% SF-B01	-	165.6	119.9	0	45.9
	0.05% SF-B01	150.4	166.5	124.3	13.0	41.5
	0.10% SF-B01	150.4	165.9	126.1	21.9	41.6
	0.25% SF-B01	150.7	166.2	126.6	17.4	42.0
	0.50% SF-B01	150.9	166.4	127.3	19.2	41.6
Injection molded, 3 mm	iPP	-	167.9	116.9	0	36.6
	0.01% NU-100	-	167.7	117.6	0	46.6
	0.05% NU-100	150.8	167.3	124.9	0.8	41.9
	0.10% NU-100	151.1	166.5	126.2	0.7	42.7
	0.25% NU-100	-	166.0	126.9	0	41.9
	0.50% NU-100	-	167.1	128.1	0	48.0
	0.01% SF-B01	-	167.0	117.9	0	43.3
	0.05% SF-B01	-	166.2	125.3	0	42.8
	0.10% SF-B01	151.2	166.4	124.9	2.2	40.3
	0.25% SF-B01	-	165.8	126.6	0	43.3
	0.50% SF-B01	-	167.0	127.4	0	42.9

**Table 2:** The thermal properties of iPP and the nucleated samples obtained during the second heating.

	Sample	*T_mβ_* [°C]	*T_mα_* [°C]	*Φ* [%]	*X_c_* [%]
Compression molded	iPP	-	162.8	0	39.2
	0.01% NU-100	-	163.1	0	52.1
	0.05% NU-100	150.7	167.6	76.2	50.1
	0.10% NU-100	151.5	167.5	75.9	49.3
	0.25% NU-100	151.9	167.6	77.8	50.0
	0.50% NU-100	151.3	167.7	75.9	50.2
	0.01% SF-B01	-	162.8	0	52.7
	0.05% SF-B01	151.6	167.6	77.6	50.5
	0.10% SF-B01	151.9	167.5	76.3	50.8
	0.25% SF-B01	152.0	167.6	78.8	50.2
	0.50% SF-B01	152.5	167.7	73.7	48.6
Injection molded, 1 mm	iPP	-	164.0	0	41.6
	0.01% NU-100	-	163.8	0	53.3
	0.05% NU-100	151.6	167.0	70.9	55.0
	0.10% NU-100	151.8	167.7	73.8	55.1
	0.25% NU-100	152.2	167.6	71.1	55.0
	0.50% NU-100	152.1	167.5	71.8	55.0
	0.05% SF-B01	-	164.0	0	52.5
	0.05% SF-B01	151.1	167.7	78.6	49.6
	0.10% SF-B01	151.7	167.5	78.6	50.2
	0.25% SF-B01	151.9	167.4	78.9	50.8
	0.50% SF-B01	152.2	167.5	78.8	51.9
Injection molded, 3 mm	iPP	-	163.3	0	40.8
	0.01% NU-100	-	163.9	0	52.9
	0.05% NU-100	151.7	167.6	77.2	50.3
	0.10% NU-100	152.0	167.6	79.3	49.9
	0.25% NU-100	152.3	167.6	79.4	50.4
	0.50% NU-100	151.6	167.0	79.3	55.4
	0.01% SF-B01	-	163.4	0	48.1
	0.05% SF-B01	151.5	167.4	78.0	51.0
	0.10% SF-B01	151.6	167.9	81.0	47.9
	0.25% SF-B01	152.2	167.5	78.2	50.83
	0.50% SF-B01	152.1	167.4	78.4	49.3

## Data Availability

The data presented in this study are available on request from the corresponding author.

## Supplementary Materials

The following supporting information can be downloaded at: https://www.mdpi.com/article/10.3390/ma16103627/s1, Figure S1: The DSC thermograms obtained during the second heating of the samples nucleated with different amounts of NU-100 and SF-B01 processed by compression molding (a, b) and injection molding into 3 mm mold (c, d); Figure S2: The DSC thermograms obtained during cooling of the samples nucleated with different amounts of NU-100 and SF-B01 processed by compression molding (a, b) and injection molding into 3 mm mold (c, d).

## References

[1] Jubinville D., Esmizadeh E., Tzoganakis C., Mekonnen T. (2021). Thermo-Mechanical Recycling of Polypropylene for the Facile and Scalable Fabrication of Highly Loaded Wood Plastic Composites. Compos. Part B Eng..

[2] Strömberg E., Karlsson S. (2009). The Design of a Test Protocol to Model the Degradation of Polyolefins during Recycling and Service Life. J. Appl. Polym. Sci..

[3] (2022). Plastics—The Facts 2022.

[4] Papageorgiou D.G., Chrissafis K., Bikiaris D.N. (2015). β-Nucleated Polypropylene: Processing, Properties and Nanocomposites. Polym. Rev..

[5] Gahleitner M. (2020). Are Polyolefins Outdated?. Express Polym. Lett..

[6] Bora R.R., Wang R., You F. (2020). Waste Polypropylene Plastic Recycling toward Climate Change Mitigation and Circular Economy: Energy, Environmental, and Technoeconomic Perspectives. ACS Sustain. Chem. Eng..

[7] Pukánszky B., Mudra I., Staniek P. (1997). Relation of Crystalline Structure and Mechanical Properties of Nucleated Polypropylene. J. Vinyl Addit. Technol..

[8] Avalos-Belmontes F., Ramos-deValle L.F., Espinoza-Martínez A.B., Martínez-Colunga J.G., Ramírez-Vargas E., Sánchez-Valdés S., Ortíz-Cisneros J.C., Martínez-Segovia E.E., Beltrán-Ramírez F.I. (2016). Effect of Different Nucleating Agents on the Crystallization of Ziegler-Natta Isotactic Polypropylene. Int. J. Polym. Sci..

[9] Tenma M., Mieda N., Takamatsu S., Yamaguchi M. (2008). Structure and Properties for Transparent Polypropylene Containing Sorbitol-Based Clarifier. J. Polym. Sci. Part B Polym. Phys..

[10] Tenma M., Yamaguchi M. (2007). Structure and Properties of Injection-Molded Polypropylene with Sorbitol-Based Clarifier. Polym. Eng. Sci..

[11] Liu X., Liu X., Li Y., Zhang Y., Xie X., Li K., Chen Z., Zhang L., Tang Z., Liu Z. (2020). Nanoengineering of Transparent Polypropylene Containing Sorbitol-Based Clarifier. J. Polym. Res..

[12] Farahani M., Jahani Y. (2021). An Approach for Prediction Optimum Crystallization Conditions for Formation of Beta Polypropylene by Response Surface Methodology (RSM). Polym. Test..

[13] Luijsterburg B., Jobse P., Hermida Merino D., Peijs T., Goossens H. (2014). Solid-State Drawing of β-Nucleated Polypropylene: Effect of Additives on Drawability and Mechanical Properties. J. Polym. Sci. Part B Polym. Phys..

[14] Nitta K., Takashima T. (2020). Tensile Properties in β-Modified Isotactic Polypropylene. Polypropylene—Polymerization and Characterization of Mechanical and Thermal Properties.

[15] Romankiewicz A., Sterzynski T., Brostow W. (2004). Structural characterization of α- and β-nucleated isotactic polypropylene. Polym. Int..

[16] Obadal M., Čermák R., Raab M., Verney V., Commereuc S., Fraïsse F. (2005). Structure Evolution of α- and β-Polypropylenes upon UV Irradiation: A Multiscale Comparison. Polym. Degrad. Stab..

[17] Cho K., Nabi Saheb D., Yang H., Kang B.-I., Kim J., Lee S.-S. (2003). Memory Effect of Locally Ordered α-Phase in the Melting and Phase Transformation Behavior of β-Isotactic Polypropylene. Polymer.

[18] Yoshida H. (1995). Dynamic Analysis of the Melting Behavior of Polymers Showing Polymorphism Observed by Simultaneous DSC/X-Ray Diffraction Measurements. Thermochim. Acta.

[19] Dragaun H., Hubeny H., Muschik H. (1977). Shear-Induced β-Form Crystallization in Isotactic Polypropylene. J. Polym. Sci. Polym. Phys. Ed..

[20] Zhang B., Chen J., Ji F., Zhang X., Zheng G., Shen C. (2012). Effects of Melt Structure on Shear-Induced β-Cylindrites of Isotactic Polypropylene. Polymer.

[21] Bednarek W.H., Paukszta D., Szostak M., Szymańska J. (2021). Fundamental Studies on Shear-Induced Nucleation and Beta-Phase Formation in the Isotactic Polypropylene—Effect of the Temperature. J. Polym. Res..

[22] Yue Y., Feng J. (2019). Structure Evolution upon Heating and Cooling and Its Effects on Nucleation Performance: A Review on Aromatic Amide Β-nucleating Agents for Isotactic Polypropylene. Polym. Cryst..

[23] Nie S., Zhong J.-R., Li Y., Zhang Y.-F. (2023). Effect of Polyacrylic Salt Nucleating Agents on the Properties of Isotactic Polypropylene. J. Therm. Anal. Calorim..

[24] Xiang J., Li Y., Zhong J.-R., Lu C.-H., Zhang Y.-F. (2023). Influence of Chemical Structures of Bisamide Nucleating Agents on the Crystallization Behavior and Properties of Isotactic Polypropylene. J. Therm. Anal. Calorim..

[25] Liu L., Zhao Y., Zhang C., Dong Z., Wang K., Wang D. (2021). Morphological Characteristics of β-Nucleating Agents Governing the Formation of the Crystalline Structure of Isotactic Polypropylene. Macromolecules.

[26] Qin W., Liu K., Xin Z., Ling H., Zhou S., Zhao S. (2020). Zinc Pimelate as an Effective β -nucleating Agent for Isotactic Polypropylene at Elevated Pressures and under Rapid Cooling Rates. Polym. Cryst..

[27] Zhou Z., Cui L., Zhang Y., Zhang Y., Yin N. (2008). Isothermal Crystallization Kinetics of Polypropylene/POSS Composites. J. Polym. Sci. Part B Polym. Phys..

[28] Fu B.X., Yang L., Somani R.H., Zong S.X., Hsiao B.S., Phillips S., Blanski R., Ruth P. (2001). Crystallization Studies of Isotactic Polypropylene Containing Nanostructured Polyhedral Oligomeric Silsesquioxane Molecules under Quiescent and Shear Conditions. J. Polym. Sci. Part B Polym. Phys..

[29] Pracella M., Chionna D., Fina A., Tabuani D., Frache A., Camino G. (2006). Polypropylene-POSS Nanocomposites: Morphology and Crystallization Behaviour. Macromol. Symp..

[30] Niemczyk A., Dziubek K., Sacher-Majewska B., Czaja K., Dutkiewicz M., Marciniec B. (2016). Study of Thermal Properties of Polyethylene and Polypropylene Nanocomposites with Long Alkyl Chain-Substituted POSS Fillers. J. Therm. Anal. Calorim..

[31] Durmus A., Kasgoz A., Ercan N., Akın D., Şanlı S. (2012). Effect of Polyhedral Oligomeric Silsesquioxane (POSS) Reinforced Polypropylene (PP) Nanocomposite on the Microstructure and Isothermal Crystallization Kinetics of Polyoxymethylene (POM). Polymer.

[32] Fina A., Tabuani D., Frache A., Camino G. (2005). Polypropylene–Polyhedral Oligomeric Silsesquioxanes (POSS) Nanocomposites. Polymer.

[33] Herc A.S., Bojda J., Nowacka M., Lewiński P., Maniukiewicz W., Piorkowska E., Kowalewska A. (2020). Crystallization, Structure and Properties of Polylactide/Ladder Poly(Silsesquioxane) Blends. Polymer.

[34] Czarnecka-Komorowska D., Sterzynski T. (2018). Effect of Polyhedral Oligomeric Silsesquioxane on the Melting, Structure, and Mechanical Behavior of Polyoxymethylene. Polymers.

[35] Niemczyk A., Dziubek K., Czaja K., Szatanik R., Szolyga M., Dutkiewicz M., Marciniec B. (2016). Polypropylene/Polyhedral Oligomeric Silsesquioxane Nanocomposites—Study of Free Volumes, Crystallinity Degree and Mass Flow Rate. Polimery.

[36] Barczewski M., Czarnecka-Komorowska D., Andrzejewski J., Sterzyński T., Dutkiewicz M., Dudziec B. (2013). Processing Properties of Thermoplastic Polymers Modified by Polyhedral Oligomeric Silsesquioxanes (POSS). Polimery.

[37] Dobrzyńska-Mizera M., Dutkiewicz M., Sterzyński T., Di Lorenzo M.L. (2016). Polypropylene-Based Composites Containing Sorbitol-Based Nucleating Agent and Siloxane-Silsesquioxane Resin. J. Appl. Polym. Sci..

[38] Mao H., Liu Y., Liu W., Nie M., Wang Q. (2017). Investigation of Crystallisation and Interfacial Nature of Polyhedral Oligomeric Silsesquioxane/Polypropylene Composites in the Presence of β-Nucleating Agent. Plast. Rubber Compos..

[39] Zhang X., Zhao S., Meng X., Xin Z. (2020). The Mechanical Properties, Crystallization and Rheological Behavior of Isotactic Polypropylene with Nucleating Agent Supported on Polyhedral Oligomeric Silsesquioxanes (POSS). J. Polym. Res..

[40] Roy S., Scionti V., Jana S.C., Wesdemiotis C., Pischera A.M., Espe M.P. (2011). Sorbitol–POSS Interactions on Development of Isotactic Polypropylene Composites. Macromolecules.

[41] Roy S., Lee B.J., Kakish Z.M., Jana S.C. (2012). Exploiting POSS–Sorbitol Interactions: Issues of Reinforcement of Isotactic Polypropylene Spun Fibers. Macromolecules.

[42] Barczewski M., Zdanowicz M., Mysiukiewicz O., Dobrzyńska-Mizera M., Dudziec B. (2023). Effect of Tetrasilanolphenyl Silsesquioxane on Properties of Sorbitol Derivative-Nucleated Polypropylene Cast Films. Plast. Rubber Compos..

[43] Dobrzyńska-Mizera M., Barczewski M., Dudziec B., Sterzyñski T. (2013). Influence of the Cooling Rate on the Non-Isothermal Crystallization of Isotactic Polypropylene Modified with Sorbitol Derivative and Silsesquioxane. Polimery.

[44] Barczewski M., Dobrzyńska-Mizera M., Dudziec B., Sterzyński T. (2014). Influence of a Sorbitol-Based Nucleating Agent Modified with Silsesquioxanes on the Non-Isothermal Crystallization of Isotactic Polypropylene. J. Appl. Polym. Sci..

[45] Gao J., Cao X., Zhang C., Hu W. (2013). Non-Isothermal Crystallization Kinetics of Polypropylene/MAP-POSS Nanocomposites. Polym. Bull..

[46] Barczewski M., Dobrzyńska-Mizera M., Dutkiewicz M., Szołyga M. (2016). Novel Polypropylene β -Nucleating Agent with Polyhedral Oligomeric Silsesquioxane Core: Synthesis and Application. Polym. Int..

[47] Chvátalová L., Navrátilová J., Čermák R., Raab M., Obadal M. (2009). Joint Effects of Molecular Structure and Processing History on Specific Nucleation of Isotactic Polypropylene. Macromolecules.

[48] Menyhárd A. (2007). Crystallization and Melting Characteristics and Supermolecular Structure of the β-Modification of Isotactic Polypropylene and Its Multi-Component Systems. Ph.D. Thesis.

[49] Fina A., Tabuani D., Carniato F., Frache A., Boccaleri E., Camino G. (2006). Polyhedral Oligomeric Silsesquioxanes (POSS) Thermal Degradation. Thermochim. Acta.

[50] Banasiak A., Sterzyński T. (2004). Assessment of a Flow of a Polymer, Filled with Lamellar Filler as a Marker, in an Injection Mold. Polimery.

[51] Li J.X., Cheung W.L. (1998). On the Deformation Mechanisms of β-Polypropylene: 1. Effect of Necking on β-Phase PP Crystals. Polymer.

[52] Ding C., Wu G.-G., Zhang Y., Yang Y., Yin B., Yang M.-B. (2019). Effect of Surfactant Assisted β-Nucleating Agent Self-Assembly on the Crystallization of Polypropylene. Polymer.

[53] Varga J., Karger-Kocsis J. (1996). Rules of Supermolecular Structure Formation in Sheared Isotactic Polypropylene Melts. J. Polym. Sci. Part B Polym. Phys..

[54] Bai H., Luo F., Zhou T., Deng H., Wang K., Fu Q. (2011). New Insight on the Annealing Induced Microstructural Changes and Their Roles in the Toughening of β-Form Polypropylene. Polymer.

[55] Luo B., Li H., Zhang W., Zhou C., Li J., Lu C., He X., Jiang S. (2017). Mechanistic Insights into the Shear Effects on Isotactic Polypropylene Crystallization Containing β-Nucleating Agent. Chinese J. Polym. Sci..

[56] Ma Y., Xin M., Xu K., Chen M. (2013). A Novel β-Nucleating Agent for Isotactic Polypropylene. Polym. Int..

[57] Nezbedova E., Pospisil V., Bohaty P., Vlach B. (2001). Fracture Behaviour Ofβ-Polypropylene as a Function of Processing Conditions. Macromol. Symp..

[58] Huo H., Jiang S., An L., Feng J. (2004). Influence of Shear on Crystallization Behavior of the β Phase in Isotactic Polypropylene with β-Nucleating Agent. Macromolecules.

[59] Stern C., Frick A., Weickert G. (2007). Relationship between the Structure and Mechanical Properties of Polypropylene: Effects of the Molecular Weight and Shear-Induced Structure. J. Appl. Polym. Sci..

[60] Zhang C., Wang B., Yang J., Ding D., Yan X., Zheng G., Dai K., Liu C., Guo Z. (2015). Synergies among the Self-Assembled β-Nucleating Agent and the Sheared Isotactic Polypropylene Matrix. Polymer.

[61] Pantani R., Coccorullo I., Volpe V., Titomanlio G. (2010). Shear-Induced Nucleation and Growth in Isotactic Polypropylene. Macromolecules.

[62] Luo F., Wang K., Ning N., Geng C., Deng H., Chen F., Fu Q., Qian Y., Zheng D. (2011). Dependence of Mechanical Properties on β-Form Content and Crystalline Morphology for β-Nucleated Isotactic Polypropylene. Polym. Adv. Technol..

[63] Varga J., Menyhárd A. (2007). Effect of Solubility and Nucleating Duality of N, N ‘-Dicyclohexyl-2,6-Naphthalenedicarboxamide on the Supermolecular Structure of Isotactic Polypropylene. Macromolecules.

[64] Karger-Kocis J., Friedrich K. (1989). Effect of Skin-Core Morphology on Fatigue Crack Propagation in Injection Moulded Polypropylene Homopolymer. Int. J. Fatigue.

[65] Kersch M., Pischke L., Schmidt H.-W., Altstädt V. (2014). Influence of Trisamide-Based Additives on the Morphological and Mechanical Properties of Isotactic Polypropylene. Polymer.

