# Supplementary material for: Effect of Shear Stress during Processing on Structure, Morphology, and Properties of Isotactic Polypropylene Nucleated with Silsesquioxane-Based β-Nucleating Agent
Source: Materials (Basel). 2023 May 9;16(10):3627. doi: 10.3390/ma16103627 (PMC10224164; doi:10.3390/ma16103627)
Supplement: Supplementary file 1 [file materials-16-03627-s001.zip › materials-2351934-supplementary.docx]

Supplementary data


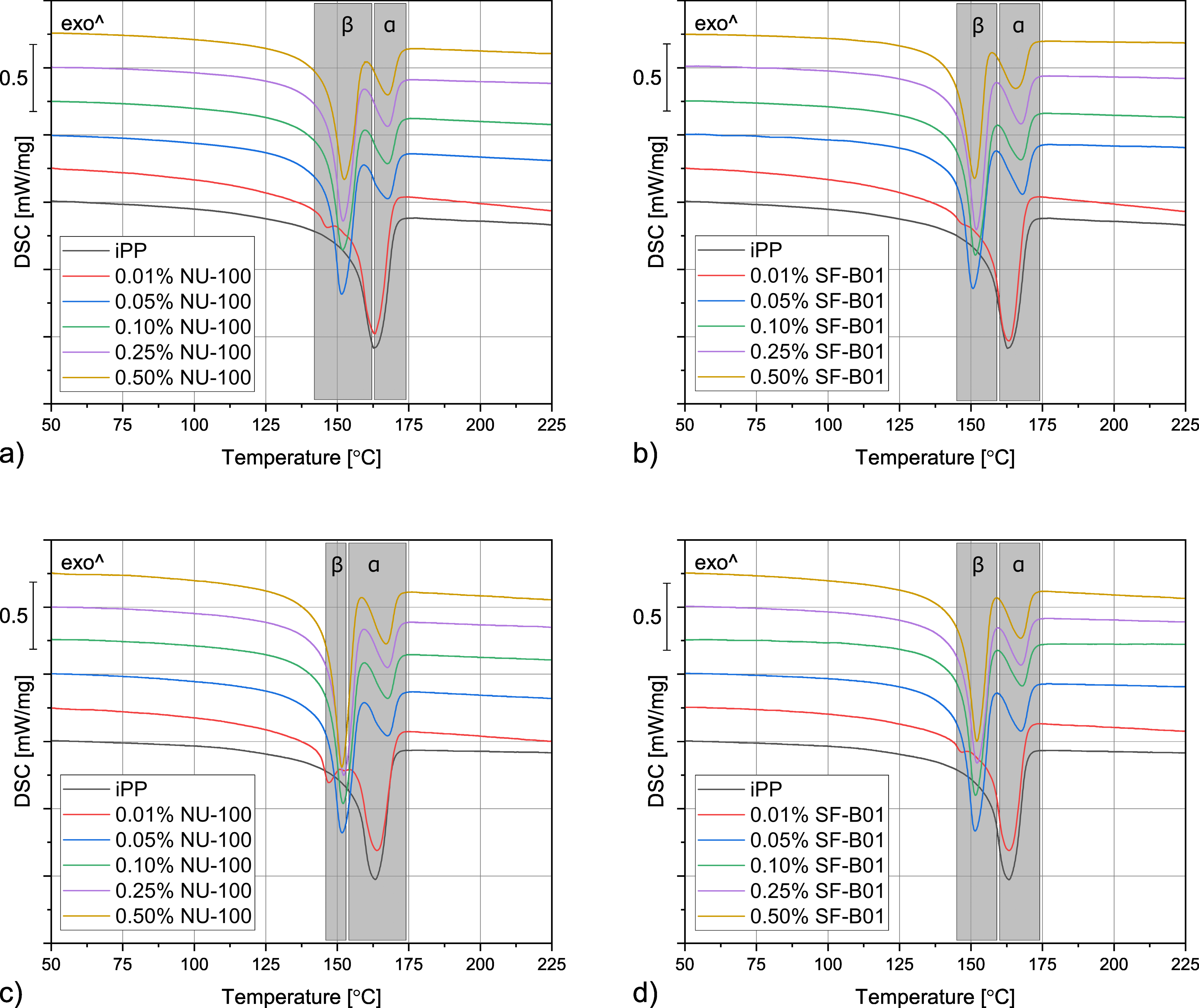


**Figure S1.** The DSC thermograms obtained during the second heating of the samples nucleated with different amounts of NU-100 and SF-B01 processed by compression molding (**a**,**b**) and injection molding into 3 mm mold (**c**,**d**).


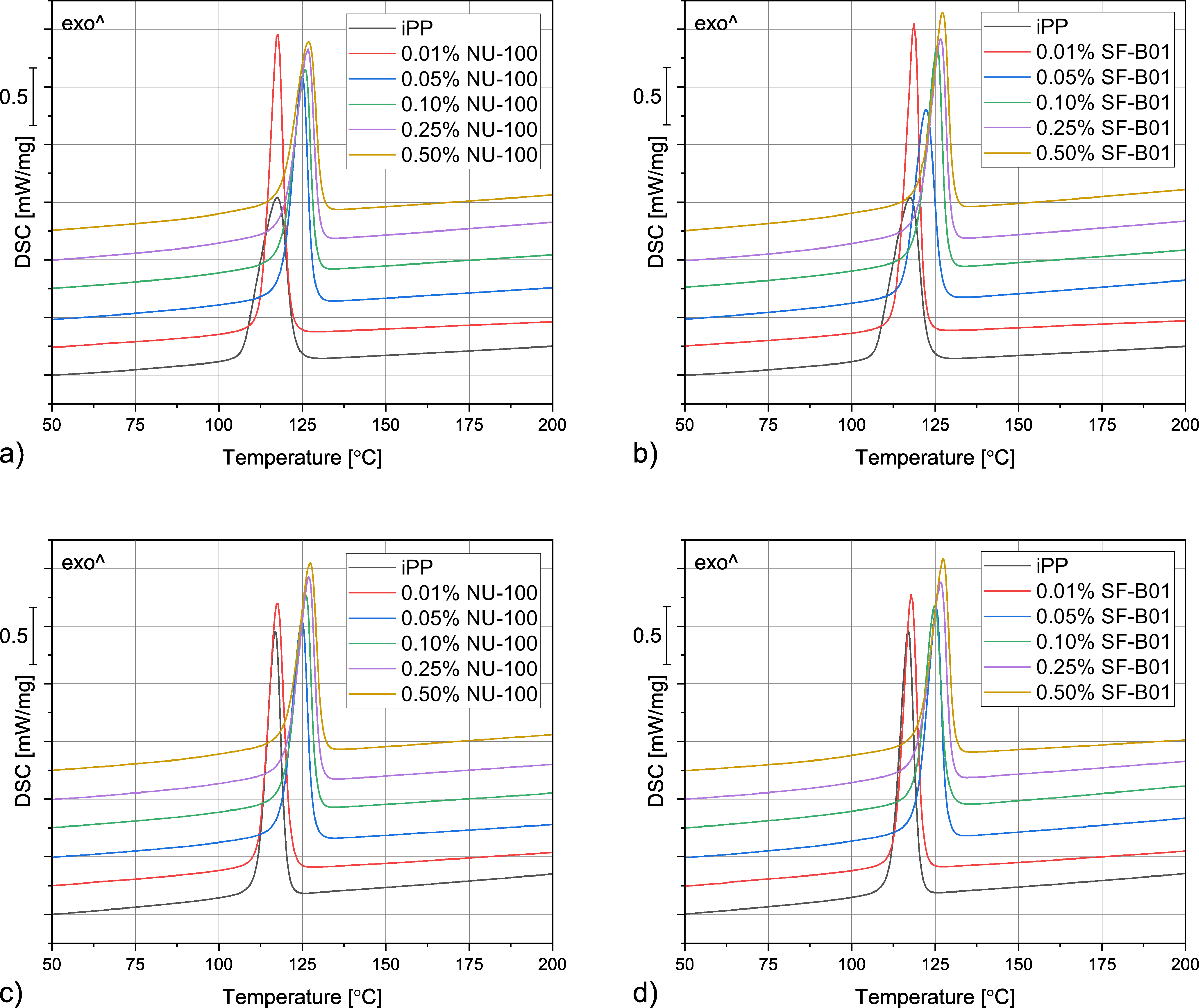


**Figure S2.** The DSC thermograms obtained during cooling of the samples nucleated with different amounts of NU-100 and SF-B01 processed by compression molding (**a**,**b**) and injection molding into 3 mm mold (**c**,**d**).
